# El Niño-Southern Oscillation, local weather and occurrences of dengue virus serotypes

**DOI:** 10.1038/srep16806

**Published:** 2015-11-19

**Authors:** Xiaodong Huang, Archie C.A. Clements, Gail Williams, Gregor Devine, Shilu Tong, Wenbiao Hu

**Affiliations:** 1 School of Public Health and Social Work, Institute of Health and Biomedecal Innovation, Queensland University of Technology, Brisbane, Queensland, Australia; 2 Research School of Population Health, The Australian National University, Canberra, ACT, Australia; 3 School of Public Health, The University of Queensland, Brisbane, Queensland, Australia; 4 Mosquito Control Laboratory, QIMR Berghofer Medical Research Institute, Brisbane, Queensland, Australia

## Abstract

Severe dengue fever is usually associated with secondary infection by a dengue virus (DENV) serotype (1 to 4) that is different to the serotype of the primary infection. Dengue outbreaks only occur following importations of DENV in Cairns, Australia. However, the majority of imported cases do not result in autochthonous transmission in Cairns. Although DENV transmission is strongly associated with the El Niño-Southern Oscillation (ENSO) climate cycle and local weather conditions, the frequency and potential risk factors of infections with the different DENV serotypes, including whether or not they differ, is unknown. This study used a classification tree model to identify the hierarchical interactions between Southern Oscillation Index (SOI), local weather factors, the presence of imported serotypes and the occurrence of the four autochthonous DENV serotypes from January 2000–December 2009 in Cairns. We found that the 12-week moving average of SOI and the 2-week moving average of maximum temperature were the most important factors influencing the variation in the weekly occurrence of the four DENV serotypes, the likelihoods of the occurrence of the four DENV serotypes may be unequal under the same environmental conditions, and occurrence may be influenced by changes in global and local environmental conditions in Cairns.

Dengue, a disease caused by the dengue virus (DENV), is the most important arbovirus in tropical and subtropical regions^1^. The number of dengue cases globally has increased 30-fold in the past five decades^2^. Infection with a specific dengue serotype causes life-long homologous immunity on first serotype-specific infection and short-lived cross-immunity^1^,^3^,^4^,^5^. People subsequently infected by heterologous serotypes are at higher risk of dengue haemorrhagic fever and dengue shock syndrome, in which the presence of antibodies of heterologous serotypes might increase replication of DENV^6^. Historically, DENV has been believed to include four serotypes (DENV-1 to 4) with a fifth serotype recently discovered, potentially further complicating dengue transmission control and disease prevention, particularly with regards to the development of a dengue vaccine^7^. Hence, there is an urgent need to fully understand the transmission dynamics of DENV serotypes.

Previous studies have demonstrated that DENV transmission is strongly associated with the El Niño-Southern Oscillation (ENSO) climate cycle and local weather conditions^8^,^9^,^10^,^11^,^12^, and that the risk of occurrence of dengue epidemics has varied according to the strength of ENSO^13^,^14^. Moreover, the effects of environmental factors on dengue transmission at different spatial scales have differed, and display complex nonlinear dynamics^11^,^13^,^14^. Some studies also demonstrated that imported dengue cases could initiate local dengue outbreaks only under suitable local weather conditions^9^,^15^. Nevertheless, previous studies have aggregated data on all four DENV serotypes. Few studies have paid attention to the effects of weather factors on DENV^16^. It is necessary to investigate the association between DENV displacement and meteorology to assess the risk of severity of local dengue epidemics^15^,^16^. In addition, Co-circulation of the four DENV serotypes has been reported widely and has increased in many regions in the world^17^. A study in Singapore showed that importation of different DENV serotypes displayed at various transmission levels and a serotype-specific outbreak often has been replaced subsequently by other serotypes^18^. Although varying predominant DENV serotypes by year from 1973 to 1999 in Thailand was reported^19^ and transmission dynamics of individual DENV serotypes have been widely described and related to climate, human behaviours and herd immunity^20^,^21^,^22^, the impact of these factors on transmission of different DENV serotypes is still unclear^21^. The dynamics of DENV are complex, involving a vector component, herd immunity, human behaviours and environmental factors^23^. The majority of imported DENV cases do not result in autochthonous transmission in non-endemic regions^24^,^25^ and little is known about whether there is a differential risk based on specific imported serotype, particularly in non-endemic areas. Uncertainty remains regarding the effects of global and local environmental factors on comparative occurrence of different DENV serotypes^26^.

Currently, dengue transmission in Australia only occurs in the north-east of Queensland, triggered by viraemic international travellers. In that region, Cairns experiences the highest number of locally acquired dengue fever cases^27^. The frequency of dengue outbreaks and the number of imported dengue cases have increased in the past two decades in north Queensland. The hyperendemic Asia-Pacific region, where all four DENV serotypes have circulated^28^,^29^,^30^, is the main source of imported dengue throughout the year in Cairns^31^.

Our previous study assessed the predictors of dengue fever incidence in the Cairns region, finding that temperature and rainfall made a contribution to local dengue fever disease transmission^9^,^10^. However, it remains unclear whether environmental factors affect the occurrence of autochthonous cases caused by specific DENV serotypes^26^. Understanding the drivers of occurrence of different DENV serotypes is important to developing future vaccination programmes, current control strategies and ongoing dengue risk assessments relating to sequential infections with different serotypes.

## Results

### Associations between autochthonous, reported imported DENV serotypes and SOI

**Figure 1:**
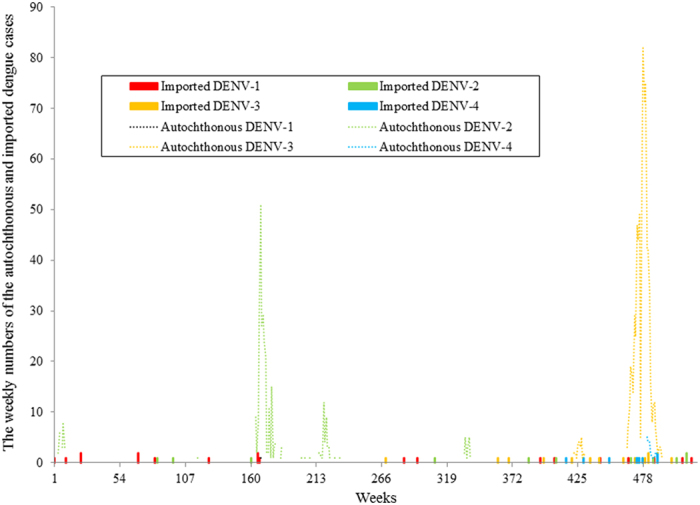
The temporal patterns of the weekly numbers of autochthonous and imported dengue cases caused by the four DENV serotypes during 1^st^ January 2000 and 31^st^ December 2009 in Cairns, Queensland, Australia.

**Figure 2:**
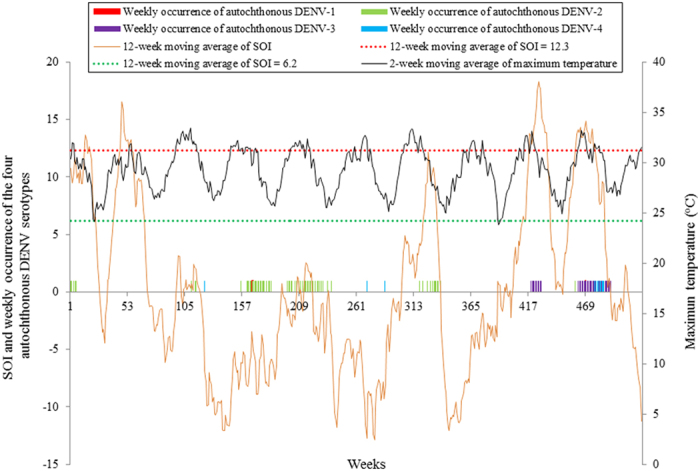
The temporal patterns of the weekly occurrence of the four autochthonous DENV serotypes, 12-week moving average of SOI and 2-week moving average of maximum temperature during 1^st^ January 2000 and 31^st^ December 2009 in Cairns, Queensland, Australia.

**Figure 3:**
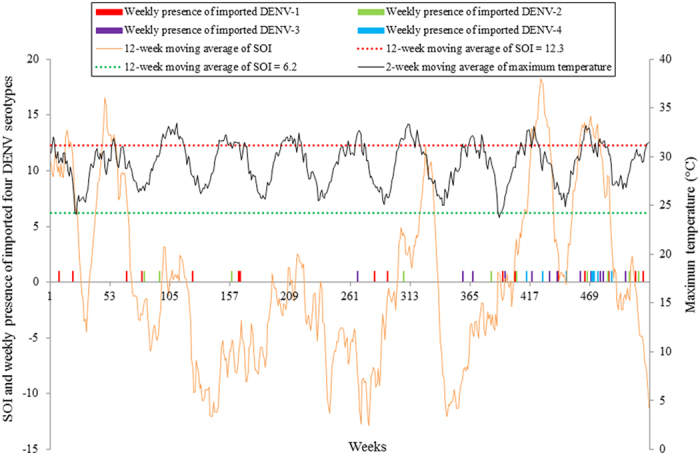
The temporal patterns of the weekly presence of reported imported four DENV serotypes, 2-week moving average of maximum temperature and 12-week moving average of SOI during 1^st^ January 2000 and 31^st^ December 2009 in Cairns, Queensland, Australia.

Figure 1 shows the weekly numbers of the reported autochthonous and imported cases for the four DENV serotypes between 1^st^ January 2000 and 31^st^ December 2009. The numbers of autochthonous cases caused by DENV-2 and -3 were much larger than DENV-1 and -4. Large outbreaks of DENV-2 were observed during 2003 and 2004 and a large outbreak of DENV-3 occurred in 2008 and 2009. Figure 1 also showed that many importations did not successfully result in an expectation outbreak. The weekly occurrence of autochthonous cases caused by DENV-2 and -3 were the most frequently observed autochthonous serotypes in the study (Fig. 2). There were only three weekly occurrences of autochthonous DENV-1 during the study period. The occurrence of autochthonous cases caused by the four DENV serotypes appeared to fluctuate randomly over the study period. Sixty-seven percent of the weekly occurrence of autochthonous DENV-1 and 57% of the weekly occurrence of autochthonous DENV-2 were observed when the values of the 12-week moving average of SOI were negative. All the weekly occurrence of autochthonous DENV-3 and 75% of the weekly occurrence of autochthonous DENV-4 were found when the values of the 12-week moving average of SOI were positive. Figure 3 showed that 53% of the weekly presence of reported imported DENV-1 and 60% of the weekly presence of reported imported DENV-2 were observed when the values of the 12-week moving average of SOI were negative. Sixty-seven percent of the weekly presence of reported imported DENV-3 and 88% of the weekly presence of reported imported DENV-4 were detected when the values of the 12-week moving average of SOI were positive. Figure 3 also showed that the diversity and number of imported DENV serotypes increased during the study period. DENV-1 was the most frequently imported DENV serotype in Cairns.

### Classification tree analysis

**Figure 4:**
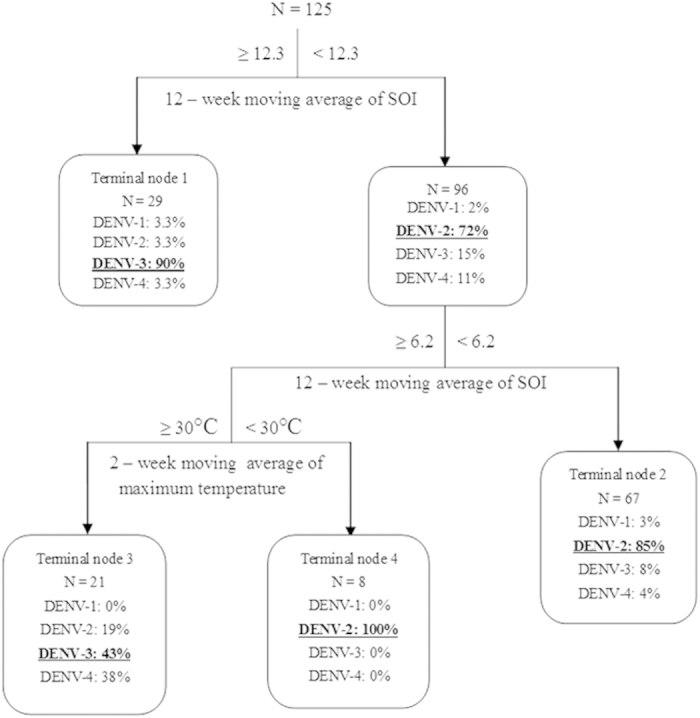
The classification tree for the weekly occurrence of autochthonous dengue cases caused by the four DENV serotypes and global/local environmental factors between 1^st^ January 2000 and 31^st^ December 2009 in Cairns, Queensland, Australia. N is the total number of the weekly occurrence of the four autochthonous DENV serotypes in each node.

The misclassification rate of the classification tree model was 19.8% in the study. After accounting for the presence of reported imports of all four DENV serotypes, the first classifying factor in the classification tree model was SOI (Fig. 4). This indicates that the 12-week moving average of SOI was the most important factor influencing the variation in the weekly occurrence of autochthonous cases caused by specific DENV serotypes. There was 90% chance of the weekly occurrence of autochthonous cases caused by DENV-3 when the 12-week moving average of SOI was ≥12.3 (Fig. 4: Terminal node 1); and there was an 85% chance of the occurrence of autochthonous cases caused by DENV-2 in a week when the 12-week moving average of SOI was <6.2 (Fig. 4: Terminal node 2). Moreover, when the 12-week moving average of SOI was ≥6.2 and <12.3, DENV-3 had the highest chance (43%) of occurrence when the 2-week moving average of maximum temperature was ≥30 °C (Fig. 4: Terminal node 3); only DENV-2 occurred when the 2-week moving average of maximum temperature was <30 °C (Fig. 4: Terminal node 4). Rainfall, minimum temperature and imports of the four DENV serotypes were not associated with the weekly occurrence of autochthonous cases caused by the four different DENV serotypes in the model. Finally, although the proportions of the weekly occurrences of autochthonous DENV-1 and DENV-4 were not the largest in the classification tree, 67% of the total weekly occurrences of autochthonous DENV-1 and DENV-4 fell into the terminal node 2 and the terminal node 3, respectively.

## Discussion

Cairns was selected as the study site because it is in a non-endemic region, where dengue outbreaks only occur following importations of DENV^27^. Outbreaks are triggered by incursions of imported DENV throughout the year. The dynamics of dengue outbreaks may not be strongly affected by the immune status of the human population due to limited exposure.

Imported DENV serotypes were introduced into Cairns throughout the year and local transmission of autochthonous DENV serotypes showed that new imports seldom triggered new outbreaks. DENV-1 has been the most frequently imported DENV serotype in Cairns^31^,^32^ and this was shown in this study (Fig. 3). However, the probability and the frequency of its subsequent detectable autochthonous transmission were very low. This may be because DENV-1 cases are more likely to be asymptomatic in comparison with cases caused by DENV-2 and -3^33^. Our study confirms that imported DENV serotypes do not necessarily lead to epidemic spread even in susceptible regions^24^,^25^,^26^. This may be explained by the substantial variation in virulence, human behaviour, chance events etc. and transmissions of different DENV genotypes under the same environmental conditions^34^.

The classification tree model identified that the 12-week moving average of SOI with a threshold value of 12.3 was a key determinant of occurrence of autochthonous cases caused by all four DENV serotypes. Cairns generally experiences La Niña episodes and more rainfall when SOI is >8^35^. The transmission of autochthonous DENV-3 was associated with a wetter environment as 90% of weekly occurrence occurred during La Niña episodes. Conversely, autochthonous DENV-2 presented the highest transmission risk (85%) when the 12-week moving average of SOI was <6.2, which indicated that the transmission of autochthonous DENV-2 was more associated with a hotter and dryer weather condition. It is well known that ENSO is a strong contributor to global inter-annual weather variability, ecosystem dynamics as well as human behaviour^36^. It is therefore not surprising that the strength of SOI is responsible for local ecological, temperature and rainfall variability. A DENV serotype study in Thailand has demonstrated that the four DENV serotypes have unique epidemiological characteristics and dominant DENV serotypes varied by year^19^. Temperature variation also influenced the vector efficiency of *Aedes. aegypti* for DENV-2 and significantly dominates the yearly cyclic pattern of dengue hemorrhagic fever^37^. Moreover, previous research has suggested that DENV transmission and DENV evolution involves many complex interactions, including cellular, host, environment, vector and population level^34^. The association between DENV transmission and weather condition might be changed due to virus and vector to adapt to climate variations^16^. The occurrence of different DENV serotypes might be driven by global environmental factors captured by variables such as SOI, which are likely to be good surrogates for a combination of local environmental variability, socioeconomic and human and mosquito behaviour. We suggest that global SOI appears to mirror a key threshold effect of integrated environmental conditions in determining the occurrence of specific DENV serotypes due to their different characteristics and the efficiency of virus transmission by mosquitoes in Cairns.

This study also found that the 2-week moving average of local maximum temperature played a distinct role in the transmissions of four DENV serotypes when the 12-week moving average of SOI was between 6.2 and 12.3. Typically, increasing temperature can shorten the time interval for the extrinsic incubation period, which is a product of the viral replication speed and Ae. aeg*ypti* survival^38^. This study indicated that transmission of the four DENV serotypes was different for the same global and local environmental conditions. Previous study indicated that the transmission rate of DENV-2 started to upswing during the latter half of hot-dry season and high temperature was suitable for the DENV-2 transmission by *Ae. Aegypti*^35^. Our study also evidenced that there was a higher probability of the occurrence of DENV-2 during El Niño episodes. We hypothesise that this may explain why studies at different spatial scales based on aggregated data for the four DENV serotypes have identified different effects of environment on dengue incidence. We also suggest that a new imported DENV serotype could trigger a specific outbreak only under appropriately integrated environmental conditions due to the complex process of transmission of the four DENV serotypes examined here. More research is required to investigate the association between climate factors and viral traits such as virulence indices and vector competence in both humans and mosquitoes based on different serotypes.

Some limitations of this study should be acknowledged. Firstly, the data did not capture all dengue cases due to asymptomatic or subclinical dengue cases, and incomplete data on DENV serotypes. This is likely to result in underestimation of the probability of the occurrence of autochthonous cases caused by specific DENV serotype (e.g. the small number of DENV-1 in the study). Secondly, other potential confounding variables such as social factors, vector control, population cross-immunity, mosquito populations, house construction, specific behaviours and occupation, were not available for the current study. Thirdly, failure to detect an effect of imported DENV serotypes on autochthonous DENV serotypes might have arisen from incomplete reporting of imported dengue cases to local health authorities. Finally, temporal autocorrelation of dengue cases may be a potential confounder, leading to biased estimation of effects. However, our study is a starting point to discuss the relationship between DENV serotypes and environmental factors and showed that a relationship may exist. In future research, further epidemiological investigation after considering potential confounders is needed to confirm the findings.

Although many studies have reported that dengue dynamics have fluctuated with ENSO using aggregated data on all four DENV serotypes^14^,^39^, our study presents a further investigation of the relationship between global and local environmental factors and dengue dynamics based on each of four autochthonous DENV serotypes. Our data suggest that suitable environmental conditions (i.e., rainfall and temperature) are more important than imported cases because they are key drivers of mosquito population dynamics. Importations happen sufficiently frequently that importation is not the main driver to an outbreak occurring.

## Conclusion

Twelve-week moving average of SOI and the 2-week moving average of maximum temperature may be important factors influencing the temporal distribution of the weekly occurrence of autochthonous cases caused by specific DENV serotypes in Cairns. Our findings suggest that the likelihoods of the occurrence of the four serotypes might be unequal under the same environmental conditions and vary with changes in global and local environments. Knowledge of the association between the occurrence of DENV serotypes and global climate and local environmental variability might be important for guiding improvements in allocating resources for disease prevention and control. Such interventions currently rely on vector control methods but may in the future include vaccines. Understanding the predictors of different serotypes might prove helpful in establishing optimal vaccination programmes and quantifying the risk and population health burden of severe dengue infections. Moreover, this study suggests that the role of global climate change in the transmission dynamics of the four DENV serotypes needs further work to inform future dengue control.

## Methods

### Study site and data collection

Cairns (latitude 16.55°S, longitude 145.46°E) is the second-largest city in north Queensland, and is a popular destination for domestic and international travellers. Cairns experiences a tropical climate with wet and dry seasons throughout the year. The highest and lowest annual mean temperatures are approximately 31°C and 18°C, respectively^40^. Annual mean total rainfall is 2008 mm^41^. Variability of climate in Australia is often related to changes in Southern Oscillation Index (SOI). Typically, Cairns experiences a reduction in rainfall during an El Niño episode and wetter weather than usual during a La Niña episode.

Data on the numbers of daily confirmed autochthonous and imported dengue cases between 1^st^ January 2000 and 31^st^ December 2009 were obtained from Queensland Health (the Queensland State government health authority). Approximately 19.8% of dengue cases with an unknown country of origin, or unknown DENV serotype, were excluded from the analysis. Imported dengue cases were defined as confirmed patients with a travel history to dengue endemic regions in the past 12 days. Autochthonous dengue cases had not recently experienced overseas travel^27^.

Daily minimum and maximum temperature, daily relative humidity and daily rainfall for 1^st^ January 2000 to 31^st^ December 2009 were obtained from the Australian Bureau of Meteorology. In this study, the SOI was used to assess global climatic variability since SOI describes interannual climate variability and the pattern of the ENSO cycle in the Pacific Ocean. SOI is calculated from the daily fluctuations in the Seal Level Pressure difference between Tahiti and Darwin. SOI is typically used to estimate the intensity of El Niño and La Niña events (two extreme events in the ENSO cycle), and to predict trends in climate variability globally. SOI yields constant negative values for a period of months when an El Niño episode is developing. Typically, the global climate experiences an El Niño episode when SOI values are negative and a La Niña episode when the SOI is positive^42^. Daily SOI was obtained from the Queensland Government, Australia^43^.

### Statistical analysis and modelling

In this study, the number of autochthonous cases was very skew between the four DENV serotypes during the study period. Hence, we used the weekly occurrence of each of the four serotypes instead of using the weekly number of cases caused by the four serotypes in order to make the data less skewed, particularly to DENV-1 and DENV-4. Data on the weekly occurrence of the four autochthonous DENV serotypes were available for a total of 125 weeks between 1^st^ January 2000 and 31^st^ December 2009. A classification tree model was used to determine threshold effects of hierarchical relationships between environmental factors and the occurrence of the four autochthonous DENV serotypes^44^. In the model, the dependent variable was a categorical variable with four categories (i.e., DENV-1 to -4). The independent variables were the strength of the ENSO^45^,^46^, local meteorological data and importation of specific DENV serotypes, each of which has biologically plausible impact on dengue transmission due, for example, to potential impacts on extrinsic incubation period (e.g. 8–12 days) and intrinsic incubation periods (e.g. 4–10 days) in the mosquito and human^47^. Specific variables included minimum and maximum temperature, relative humidity and rainfall, at a lag of 1 week, the presence of reported imported cases of each DENV serotype at lags of 1–5 weeks, 2–4 week moving averages of local weather and 8 and 12-week moving averages of SOI.

A classification tree model was used to segment the distribution of the weekly occurrence of the four autochthonous DENV serotypes into subsets that were most likely to be associated with a particular combination of environmental conditions. The classification tree was built by repeating best splits of the environmental factors and the weekly presence of the four imported DENV serotypes in predicting the probability of the weekly occurrence of the four autochthonous DENV serotypes. The principle of impurity reduction (measured using the Gini improvement statistic) was used as the criterion for defining the best split. Cross-validation was used to select the tree size using estimated prediction errors. The ‘best’ tree is defined as having the smallest tree size and an estimated error rate within one standard error of the minimum^44^.

## Additional Information

**How to cite this article**: Huang, X. *et al.* El Niño-Southern Oscillation, local weather and occurrences of dengue virus serotypes. *Sci. Rep.* **5**, 16806; doi: 10.1038/srep16806 (2015).
